# Analysis of gene expression in the nervous system identifies key genes and novel candidates for health and disease

**DOI:** 10.1007/s10048-017-0509-5

**Published:** 2017-02-11

**Authors:** Sarah M Carpanini, Thomas M Wishart, Thomas H Gillingwater, Jean C Manson, Kim M Summers

**Affiliations:** 1 The Roslin Institute and Royal (Dick) School of Veterinary Studies, University of Edinburgh, Easter Bush, Midlothian, EH25 9RG UK; 2 Centre for Integrative Physiology, University of Edinburgh, Hugh Robson Building, 15 George Square, Edinburgh, EH8 9XD UK

**Keywords:** Mice, Neurological mutants, Neurological disorders, Transcriptome, Gene expression profiling

## Abstract

The incidence of neurodegenerative diseases in the developed world has risen over the last century, concomitant with an increase in average human lifespan. A major challenge is therefore to identify genes that control neuronal health and viability with a view to enhancing neuronal health during ageing and reducing the burden of neurodegeneration. Analysis of gene expression data has recently been used to infer gene functions for a range of tissues from co-expression networks. We have now applied this approach to transcriptomic datasets from the mammalian nervous system available in the public domain. We have defined the genes critical for influencing neuronal health and disease in different neurological cell types and brain regions. The functional contribution of genes in each co-expression cluster was validated using human disease and knockout mouse phenotypes, pathways and gene ontology term annotation. Additionally a number of poorly annotated genes were implicated by this approach in nervous system function. Exploiting gene expression data available in the public domain allowed us to validate key nervous system genes and, importantly, to identify additional genes with minimal functional annotation but with the same expression pattern. These genes are thus novel candidates for a role in neurological health and disease and could now be further investigated to confirm their function and regulation during ageing and neurodegeneration.

**Electronic supplementary material:**

The online version of this article (doi:10.1007/s10048-017-0509-5) contains supplementary material, which is available to authorized users.

## Introduction

The average lifespan of individuals in the developed world has increased dramatically over the last century, as deaths from trauma and infection have declined. The incidence of neurodegenerative diseases associated with ageing, including dementia, has risen concomitantly, bringing significant social and economic costs. However, our understanding of genetic factors controlling nervous system form and function in health and disease is far from complete. Thus, the identification of genes that control neuronal health, and elucidation of core molecular interactions that could ultimately be exploited for the development of novel therapeutic interventions, remains a major challenge.

A large proportion of genes in animals are involved in the development, differentiation, maintenance and functioning of the nervous system. For example, in *Drosophila*, 11% of annotated and predicted genes showed a specific neurological phenotype upon knockdown [1] with 336 showing a strong phenotype and 2106 showing moderate to weak phenotypes. Humans with chromosomal microdeletion or microduplication syndromes (also known as contiguous gene syndromes) frequently experience intellectual disability, indicating both the complexity of the pathways and the density of genes for neuronal development in the human genome [2, 3]. The nervous system shows a high level of transcriptional diversity. Approximately 80% of all transcripts are expressed in mammalian brain [4–7]. In one study [8], adult human brain regions expressed more than twice as many different transcripts as pancreas. Understanding the functions and interactions of the different genes expressed by cell types within the nervous system is critical if the key genetic networks modulating form and function of the mammalian nervous system are to be clarified, but many of the genes are unknown or poorly annotated and there is little idea of their function, specificity or importance.

Analysis of mouse and human immune and connective tissue gene expression data has been used previously to infer gene functions of novel genes from co-expression networks [9–14]. Numerous datasets documenting the transcriptome of the mammalian nervous system are also available. Importantly, these have revealed the complexity of networks regulating health of the nervous system and implicate roles for the wide variety of supporting (glial) cell types [15–18] including astrocytes, oligodendrocytes, microglia and connective tissue cells that make up the majority of cells in the human brain [19]. We now present an analysis of combined datasets of transcription in mouse tissues, revealing key genes and networks present in the mammalian nervous system, and review the literature concerning nervous system cell type-specific genetic markers. We identify previously unknown genes involved in regulating neuronal form and/or function which provide new targets for defining critical pathways that sustain nervous system health.

## Materials and methods

### Gene expression analysis

Public domain microarray experiments that used the Affymetrix MOE-430 microarray platform were identified in GEO DataSets (http://www.ncbi.nlm.nih.gov/gds/). A list of the datasets used in the analysis is provided in Online Resource 1. All samples were from male C57BL/6 mice at 8–10 weeks, except where stated. The raw data (.cel) files were downloaded from GEO DataSets. Quality control, normalization and probe annotation (using the most recent annotation file from Affymetrix, dated 7 October 2014) were performed using the RMA procedure [20] within the Affymetrix Expression Console software package [21]. The merged intensity file was then prepared for analysis by the network analysis tool BioLayout *Express* ^3D^. Where there were multiple samples from the same experiment, the normalized values were averaged. Probes showing low average expression (where no sample reached an intensity of ≥100 for that probe) were removed from the analysis. The dataset containing the results for the remaining probe sets was saved as a ‘.expression’ file containing a unique identifier for each row of data (in this case, gene symbol concatenated to probe set ID), followed by columns of gene annotations and finally, natural-scale normalized data values for each sample, each column of data being the averaged values derived from a different cell or tissue type.

**Fig. 1:**
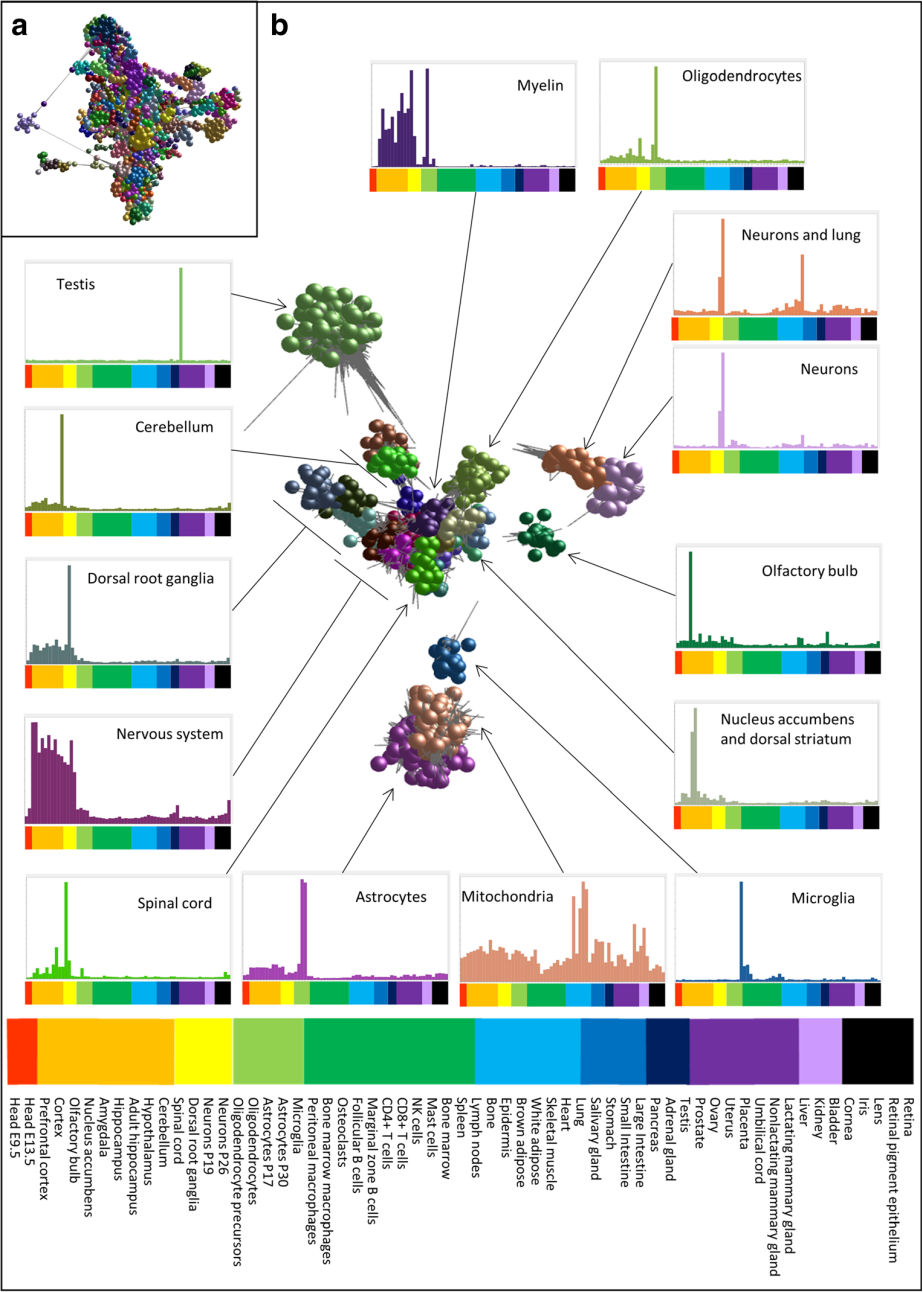
Network visualization and clustering of gene expression patterns. **a** Network graph of the main element, showing genes (spheres) and correlations between them of *r* ≥ 0.9 (*grey lines*). Nodes in clusters of genes with similar expression patterns as determined by the MCL clustering algorithm (inflation value 2.2) are shown in the same *colour*. Replicates were averaged before clustering. **b** Network graph showing only the clusters used for validation (testis, mitochondria) and nervous system clusters. Histograms surrounding the network graph show the average expression patterns of each cluster or group of related clusters. Nervous system shows average expression of six clusters; Dorsal root ganglia shows average of three clusters; Cerebellum and nucleus accumbens/dorsal striatum show average of two clusters each. *Colours of bars* in histograms are the same as the *colours of nodes* in the network graph except for the grouped clusters. *Bars below the graph* indicate the samples shown. Genes in the clusters are given in Online Resource 3. The key to the order of samples shown in histograms is shown at the *bottom* of the figure. The GEO DataSet accession numbers for all samples are given in Online Resource 1 where more information about the samples can also be found

This file was then loaded into BioLayout *Express* ^3D^ [21] and a Pearson correlation matrix calculated for each pair of probe sets on the array. A probe-to-probe analysis was performed. The network graph was laid out using a modified Fruchterman-Rheingold algorithm [22] in 3-dimensional space in which nodes representing probe sets are connected by weighted, undirected edges representing correlations between expression patterns above the selected threshold. A correlation cutoff of *r* = 0.9 was used to construct a graph containing 17,111 nodes (probe sets) and 1,836,241 edges (correlations ≥0.9). The resultant graph was large and highly structured (Fig. 1a) consisting of one large component of 12,912 nodes and >1500 smaller components (unconnected networks of correlations) of between 2 and 28 nodes. The topology of the graph contained localized areas of high connectivity and high correlation (representing groups of genes with similar profiles), determined using the Markov clustering algorithm (MCL) [23], which has been demonstrated to be one of the most effective graph-based clustering algorithms available [24]. An MCL inflation value of 2.2 was used as the basis for determining the granularity of clustering, as it has been shown to be optimal when working with highly structured expression graphs [21]. The minimum cluster size was five nodes. Clusters of greatest similarity in terms of expression were physically close to each other in the network graph. Clusters were numbered according to the number of nodes they contained, the largest cluster being designated Cluster001, and manually annotated based on the tissue(s) with greatest mean expression of probes in the cluster. A cluster was said to show tissue-specific expression for the designated tissue if the average expression of genes in the cluster in that tissue was fivefold of that in other tissues.

To validate the clusters, three additional analyses were performed, using promoter-based expression data for mouse from the FANTOM5 project, microarray data for pig available from BioGPS (http://biogps.org.org) and the initial data set supplemented by results for hippocampus from mice treated with the prion ME7. Details of these analyses are provided in supplementary methods (Online Resource 2).

### Gene function

Twenty-six expression clusters were chosen for further evaluation: 2 for validation and a further 24 for examination of gene expression in different cell types and/or regions of the nervous system. Pathway analysis was performed using Ingenuity Pathway Analysis (IPA) software [25] (http://www.ingenuity.com/products/IPA). All genes in these clusters were assessed for murine and human phenotypes using MouseMine (http://www.mousemine.org/mousemine/begin.do) [26]. For murine models, we included data for all genetic backgrounds. To prevent inconsistencies due to incomplete gene knockout, we restricted our analyses to those phenotypes associated with homozygous knockout/null mutations only. The *p* values were calculated using Bonferroni test correction against the background population with a maximum *p* value of 0.05 taken as significant. It must be noted that some genes are intensely studied and many knockout mouse models have been generated, which could potentially overinflate the statistics.

### Classification of gene annotation

Gene ontology (GO) terms [27] were included in the normalized annotated file retrieved from the Affymetrix Expression Console. Three categories were included: GO biological process, GO molecular function and GO cellular component. To assess the level of annotation for each cluster, each gene in the cluster was scored either 0 or 1 for each GO category depending on the presence (no matter how minimal) or absence of gene ontology information. An average score weighted by the size of the cluster was calculated for each cluster (maximum score of 3, if each GO category had an entry for each gene in the cluster).

## Results

### Clustering of the mouse transcriptome reveals tissue-specific expression patterns

Using the vast amount of data in the public domain, we aimed to define genes critical for influencing neuronal health and viability. We have reanalyzed 135 mouse microarray datasets from eight independent experiments. The pooled datasets represent 64 different tissue/cell types, including various brain regions, spinal cord, ganglia, astrocytes, isolated neurons, embryonic head and cell types from other organ systems (Online Resource 1). These datasets were all derived using the Affymetrix MOE-430 platform and all from male C57BL/6 mice at 8–10 weeks, unless stated (Online Resource 1). Gene expression patterns were clustered using the network analysis tool BioLayout *Express* ^3D^ to explore the relationships of the different cell and tissue types in the analysis [28]. The importance of analysis by BioLayout *Express* ^3D^ is that it clusters nodes based solely on statistical correlations of gene expression patterns and makes no assumptions about relationships between genes. Therefore, unlike many other approaches, it does not rely on published data on networks or known regulatory pathways and uses an unsupervised and model-free approach to cluster biologically derived data, in this case, normalized expression intensity levels derived from microarrays, which can highlight relationships that would otherwise be unsuspected. The algorithms underlying BioLayout *Express* ^3D^ have been explained in detail previously [21, 28].

The major element of the network graph of the probe-to probe analysis (performed as described in ‘Methods’) is displayed in Fig. 1a. The MCL clustering algorithm allocated similarly expressed genes to specific clusters [29] (different coloured nodes in Fig. 1a). The graph was explored extensively in order to understand the significance of the gene clusters and the differential gene expression patterns across the neurological tissues sampled. We determined the most common biological roles associated with genes in each Biolayout *Express* ^3D^ cluster to enable comparison of different neuronal cell and tissue types. We performed detailed analyses of each cluster for higher-order canonical pathway analysis (IPA), associated human diseases (MouseMine using data available from Online Mendelian Inheritance in Man (OMIM)) and knockout mouse phenotype (MouseMine using data available from Mouse Genome Informatics (MGI)). The unique genes present in each cluster are listed in Online Resource 3. A summary of phenotypes associated with the genes within the clusters is presented in Table 1 and full details of human disease and knockout mouse phenotype are provided in Online Resources 4 and 5, respectively.

**Table 1:** Summary of phenotypic information for genes identified in each cluster

Cell/tissue type	Description	Cluster	No. Genes	No. Genes with KO mouse models	Top phenotype (*p* value)	No. Genes with human diseases
Nervous system	General	5	144	72	Abnormal synaptic transmission (3.53 × 10^−26^)	28
		30	67	37	Abnormal nervous system physiology (1.20 × 10^−17^)	10
		53	19	7	Abnormal behavioural response to alcohol (3.85 × 10^−5^)	8
		60	21	11	Abnormal neuron physiology (0.001046)	3
		73	17	6	Abnormal CNS synaptic transmission (1.61 × 10^−9^)	0
		77	19	6		3
	Variable	59	26	12	Abnormal nervous system physiology (3.1 × 10^−6^)	1
		89	18	6	Increased circulating gastrin level (4.51 × 10^−5^)	0
		99	13	8		3
Neurons	Isolated neurons and lung	48	31	19	Abnormal vascular development (2.30 × 10^−25^)	7
	Isolated neurons	45	37	23	Increased systemic arterial blood pressure (2.28 × 10^−6^)	8
Glia	Astrocytes	6	132	70	Nervous system phenotype (2.11 × 10^−7^)	24
	Oligodendrocytes	35	50	26	Abnormal myelination (9.54 × 10^−9^)	3
	Microglia	85	21	10	Abnormal immune system physiology (6.34 × 10^−14^)	7
Nervous system regions	DRG	27	60	31	Abnormal touch/nociception (7.98 × 10^−21^)	13
		66	19	5		2
		88	12	3		0
	Cerebellum	37	45	26	Nervous system phenotype (8.0 × 10^−10^)	6
		56	18	6	Abnormal CNS synaptic transmission (1.96 × 10^−8^)	4
	Nucleus accumbens	42	39	19	Abnormal behaviour (1.70 × 10^−12^)	5
		96	14	7	Decreased susceptibility to diet-induced obesity (0.000936)	4
	Spinal cord	90	13	6		4
	Olfactory bulb	82	18	7	Abnormal facial nerve morphology (0.001383)	0
Myelin	Synthesis	91	8	4	Impaired coordination (0.000315)	1
Other	Testis	1	1627	288	Abnormal male reproductive system physiology (2.16 × 10^−125^)	103
	Mitochondria	14	87	10	Decreased embryo size (0.000874156)	32

It was important to establish that the clustering approach grouped together genes of similar function. We therefore first analysed two clusters where the highest gene expression was in tissues other than nervous system cell types. Cluster001, the largest cluster identified by BioLayout *Express* ^3D^, contained 1627 genes. These genes were strongly expressed in the testis with low expression in all other tissue types. IPA revealed that 375 genes have previously been associated with diseases affecting the reproductive system. One hundred and three genes have been associated with human disease including ciliary dyskinesia and spermatogenic failure and knockout mouse phenotype ontology enrichment reported abnormal male reproductive system physiology. Thus, the clustering analysis has grouped genes associated with reproduction, consistent with the high expression in testis. Cluster014 showed highest expression in heart, skeletal muscle and brown adipose tissues, and moderate expression in nervous system tissues. It contained 87 genes, many encoding members of each of the mitochondrial respiratory chain complexes. IPA of this cluster identified a network of genes related to oxidative phosphorylation and almost all genes had GO terms relating to mitochondria. Human diseases included deficiencies of mitochondrial complexes I, II, III, IV and V. The top associated knockout mouse phenotype was decreased embryo size, but other phenotypes included abnormal mitochondrial physiology and abnormal cellular respiration. Mitochondria regulate redox signalling, which then regulates activity of transcription factors involved in axis specification of the embryo [30], which may account for the association with size. Thus Cluster014 contains a group of genes which are involved in mitochondrial function. Comprehensive analysis of these two clusters of genes therefore showed that the clustering approach had grouped known genes of similar function and location, confirming the validity of our clustering approach.

### Distinct gene expression patterns within the nervous system

Large-scale proteomic and gene expression analysis studies have highlighted cell type-specific gene expression and regional diversity in the mammalian brain [31, 32]. We therefore analysed all clusters of genes with high expression in various nervous system tissues and cell types with the overall aim of identifying genes with minimal functional annotation but similar pattern of expression to known genes. Twenty-four clusters of genes were chosen for detailed analysis based on the following criteria:

1. The cluster contained at least eight unique genes (to provide adequate power);
2. The cluster genes were expressed in specific brain regions, neuronal tissue or glia cell types.

Six clusters contained genes highly expressed across neuronal tissues and a further three contained genes expressed at variable levels across the neuronal tissues. These clusters were located close to each other in the network graph, highlighting the similarity of their expression patterns (Fig. 1b). Many additional clusters contained genes with high expression in a specific brain region (dorsal root ganglia, nucleus accumbens and dorsal striatum, spinal cord and cerebellum). These clusters were located close to the more general neuronal clusters (more similar expression pattern) but distinct from them in the network graph. Other clusters of genes showed high expression in a single cell type (astrocytes, oligodendrocytes, microglia and isolated neurons) and were located in regions distinct from others in the analysis. We also identified one cluster of genes with high expression in both neurons and lung. In the network graph, this group was located close to the neuron cluster (Fig. 1b).

### Functions of coexpressed neuronal genes

We initially focused on Cluster005, the largest neuronal cluster. Cluster005 contained 144 genes, many encoding proteins known to be related to neuronal function, including the neuron-specific gene *Eno2* (enolase 2, gamma, neuronal) which functions in the glycolytic pathway and is used as a marker for neurons [33, 34]. Other genes in this cluster included neurotransmitter receptor components and microtubule-and synaptosome-associated proteins, with a strong representation of genes encoding proteins involved in the function of the synapse, which is increasingly accepted as key to neuronal health [35]. The genes are shown in Online Resource 3. IPA identified canonical pathways associated with signalling at the synapse (including Huntington’s disease signalling and reelin, GNRH, CDK5 and glutamate receptor signalling) and the top associated networks were cell-to-cell signalling and interaction, nervous system development and function and cellular assembly and organisation. Human disease phenotypes included autism, epilepsy and neurodegenerative disease, while the top associated knockout mouse phenotype was abnormal synaptic transmission. Human and mouse diseases are listed in Online Resources 4 and 5, respectively. Recent developments have highlighted the synapse as an early target in a broad range of neurodegenerative diseases occurring prior to neuronal pathology [35]. This largest neuronal cluster showing consistent and specific phenotypes associated with synapse function highlights the importance of the synapse in neuronal health and disease.

Five additional clusters were identified with general expression within neurons (Cluster030, 053, 060, 073 and 077). In total, these clusters contained 143 genes (Online Resource 3), all associated with cellular signalling: neuropathic pain signalling in dorsal horn neurons, (Cluster030), GABA receptor signalling (Cluster053), clathrin-mediated endocytosis signalling (Cluster060), nNOS signalling in neurons (Cluster073) and CDP-diacylglycerol biosynthesis I (Cluster077). Human diseases associated with these genes were most often forms of epilepsy but also mental retardation, spinocerebellar ataxia, Charcot-Marie-Tooth disease and spastic paraplegia (Online Resource 4). Knockout mouse models showed phenotypes including abnormal nervous system physiology, abnormal behavioural response to alcohol, abnormal neuron physiology and abnormal CNS synaptic transmission (Online Resource 5). There were also three clusters with variable expression within neuronal tissues (Cluster059, 089 and 099). IPA showed that these clusters were also all associated with signalling pathways (glutamate receptor, JAK/Stat and GABA receptor signalling). The top phenotypes in mouse models were abnormal nervous system physiology, increased circulating gastrin level and abnormalities of nerve conduction, synapses, neurodegeneration or body size. These eight clusters were all associated with neuronal signalling, emphasising the necessity of electrochemical signal transmission in maintaining neuronal health and viability.

### Functions of genes expressed in glial cell types: Cluster006, 035 and 085

In addition to neurons, the central nervous system also contains non-neuronal glial cell types. These were originally thought to provide ‘support’ to the neurons but are now seen to have critical roles in neuronal development and function [36, 37]. Glial cells have individual distinct gene profiles with astrocytes and oligodendrocytes having a transcriptome as diverse as that of neurons [15]. As a result, we included different glial cell types in our analysis (astrocytes, oligodendrocytes and microglia) and identified distinct clusters of genes associated with high expression in each of these cell types.

Cluster006 contained genes strongly expressed in astrocytes, including glial fibrillary acidic protein (*Gfap*), ion channel genes, solute carrier genes, dystrophin-related genes, ATPase genes and ubiquitin-specific peptidase. The major associated network function identified using IPA was glutamate receptor signalling, reflecting the role astrocytes play in glutamate clearance [38]. Neurological phenotypes such as migraine, deafness and ataxia were associated with mutations in these genes in humans (Online Resource 4). Knockout mouse phenotypes included nervous system phenotype and abnormal nervous system physiology (Online Resource 5). Recent studies have identified roles for astrocytes in synaptic transmission modulation, regulation of cerebral blood flow and release of ‘gliotransmitters’. Like microglia, they are involved in reactive gliosis. The association of genes in this cluster with neurological phenotypes highlights the wide array of functions astrocytes contribute to neuronal stability [39].

Cluster035 contained genes strongly expressed in oligodendroctyes including *Mag* (encoding myelin-associated glycoprotein), *Mobp* (encoding myelin-associated oligodendrocyte basic protein) and *Myrf* (encoding myelin regulatory factor) reflecting the role that oligodendrocytes play in the generation of central nervous system myelin. IPA analysis revealed the top canonical pathway to be axonal guidance signalling and Cluster035 contained three members of the semaphorin gene family involved in oligodendrocyte guidance during development [40], including the oligodendrocyte-specific semaphorin, *Sema5a* [41]. Human genes were associated with spastic paraplegia and age-related macular degeneration. Unsurprisingly, the top associated mouse knockout phenotype was abnormal myelination. In addition, we identified a small cluster of genes linked to myelin synthesis (Cluster 091) with high expression in oligodendrocytes and spinal cord and moderate expression in various brain regions. Within this cluster, knockout mouse models have been reported with the top associated canonical phenotype being impaired coordination. IPA identified the top canonical pathway for Cluster091 to be remodelling of epithelial adherens junctions, an intriguing finding given the role of E-cadherin in Schwann cell myelination [42]. Details are available in Online Resources 3, 4 and 5.

Cluster085 contained genes strongly expressed in microglia, with much lower expression in other macrophage lineages, including many cytokine genes, such as *Il10* and *Marco* (encoding macrophage receptor with collagenous structure). The majority of human diseases associated with these genes impact on the immune system, reflecting the role microglia play in the immune response. The top affected system in mouse knockouts was abnormal immune system physiology (including inflammation of specific organs, abnormal cytokine level and abnormal response to infection). Microglia are the macrophages of the CNS with a functional role in both development and maintenance of the central nervous system and recent studies have highlighted additional roles including synaptic pruning during development [43]. Microglia underlie different neurodegenerative disease processes, including lipomembranous osteodysplasia with sclerosing leukoencephalopathy (Nasu-Hakola disease) caused by mutations in *TREM2* and adult onset leukoencephalopathy with axonal spheroids and pigmented glia due to *CSF1R* haploinsufficiency (reviewed in [44]). These genes were found in a broader macrophage cluster.

### Differential functions of genes expressed in specific regions of the nervous system

Different regions of the mammalian brain have different motor, sensory and cognitive functions, and it is therefore not surprising that previous genome-wide expression studies have identified regional expression gene signatures with the cerebellum showing the greatest diversity [4, 45]. We therefore expanded our analysis to clusters of genes that showed high expression within specific brain regions. Biolayout *Express* ^*3D*^ identified three clusters associated with the dorsal root ganglion (DRG; Cluster027, 066 and 088). The largest of these clusters, Cluster027, contained the protocadherin alpha gene cluster and genes encoding cation channels, peripherin (*Prph*), peripheral myelin protein 2 (*Pmp2*) and the DRG homeobox gene (*Prrxl1*). The top associated mouse phenotype was abnormal touch/nociception. A smaller cluster, Cluster066, contained genes encoding MAP proteins (microtubule associated proteins) such as *Map1b* which is expressed in the DRG during spinal cord development and regeneration [46] with IPA identifying the top pathway to be axonal guidance signalling. Interestingly axonal guidance signalling has now been shown to be important in the mature CNS in the regulation of synaptic activity and neuronal plasticity [47].

Two clusters were associated with high gene expression in the cerebellum (Cluster037 and 056). Genes in Cluster037 included the amyotrophic lateral sclerosis gene (*Als2*) and the top IPA pathway was glutamate receptor signalling. The top associated murine phenotype was nervous system phenotype. Cluster056 contained the cadherin gene *Cdh7*, previously shown to modulate connectivity of mossy fibres in the cerebellum [48] and cation channel genes. The top canonical pathway was stimulatory G protein (Gs) signalling and knockout mouse models showed defects in CNS synaptic transmission.

Two clusters were associated with high expression in the nucleus accumbens and dorsal striatum (Clusters042 and 096) functioning in reward and decision making. The top IPA pathways were Gs signalling (Cluster042) and GABA receptor signalling (Cluster096) both functioning in neurotransmitter release and reward processing; for example, dopamine binds to a G protein-coupled receptor for Gs signalling. Phenotypes found in knockout mice included abnormal behaviour and obesity and energy expenditure. One small cluster (Cluster090) contained genes strongly expressed in the spinal cord and adult brain, including the genes for myelin oligodendrocyte glycoprotein (*Mog*) and the synaptobrevin protein (*Vamp1*). The top canonical pathway was serotonin receptor signalling, associated with development and regeneration of spinal motor neurons [49]. Cluster082 was associated with high expression in olfactory bulb. The top canonical pathway was G protein signalling mediated by the Tubby family of proteins, which play a major role during development and post-differentiation of neuronal cells (reviewed in [50]), and the top phenotype in knockout mice was abnormal facial nerve morphology.

One cluster contained genes that were highly expressed in isolated neurons (Cluster045). IPA identified the top canonical pathway to be hypoxia signalling in the cardiovascular system. Cluster045 genes have been associated with stroke, hypertension and Alzheimer’s disease in humans. The top phenotype in knockout mouse models was increased systemic arterial blood pressure indicating the importance of neuronal factors in the regulation of blood pressure, for example, through baroreceptors. For a further cluster of genes expressed within both neurons and lung (Cluster048), IPA identified the top pathway to be STAT3 signalling. Genes within this cluster included *Ndnf* (neuron-derived neurotrophic factor) which has high messenger RNA (mRNA) expression in human lung (http://www.gtexportal.org/home/gene/NDNF) and is upregulated upon ischemia [51]. Human diseases included basal ganglia calcification and venous malformations. The top phenotype for knockout mouse models was abnormal vascular development. Both clusters were also enriched for GO terms indicating extracellular localisation and involvement in cell migration.

### Tissue-specific clusters replicate in other datasets

To assess whether the clusters of genes identified as being tissue-specific were more broadly applicable, we performed two replication studies (Online Resource 2). Firstly, we used mouse data from the FANTOM5 project, which provides promoter level expression values across the transcriptome. This included astrocytes from different sources but no oligodendrocytes. The testis cluster replicated well. Of 931 testis cluster genes that were annotated in the mouse dataset, 92% were in a FANTOM5 testis cluster. We found that the majority of annotated genes in our neurological clusters were also in neurological clusters in this data set. For example, 111 genes of mouse Cluster005 (the largest nervous system cluster) were found in the FANTOM5 dataset. Fifty-nine percent of these were in the largest FANTOM5 neurological cluster and an additional 26% were in another FANTOM5 cluster with nervous system expression (e.g. cortex, cerebellum, olfactory brain, etc.). The lack of oligodendrocytes and additional region specific astrocyte samples meant that some clusters did not replicate, as might be expected. We then looked at a published dataset for gene expression based on microarrays in the pig [13] (http://biogps.org). In this dataset, many of the neurological tissues were missing and there were no primary glial cells (Online Resource 2). In addition, fewer genes were annotated in the pig data set so many of the mouse genes could not be found. Again, the testis cluster replicated well with 54% of genes (that were found in both datasets) in the main pig testis cluster and 58% overall in clusters with testis specific expression. In addition, 18% of the genes from the mouse testis cluster were found in a pig fallopian tube cluster. Sixty-six percent of the genes from the mouse Cluster005 that could be found in the pig dataset were in the pig cortex cluster while others were in the spinal cord and cerebellum clusters. In addition, 67% of genes in mouse cluster 30 (nervous system) were also in the pig cortex cluster. Given the limited tissue sets available for the pig and the differences in annotation and naming conventions between pig and mouse, with many more unannotated pig genes, this result shows a reassuring level of concordance and we believe validates the clustering approach as a means to find genes with shared expression patterns and hence assign function to unannotated genes.

### The level of annotation for genes within expression pattern clusters varies

**Fig. 2:**
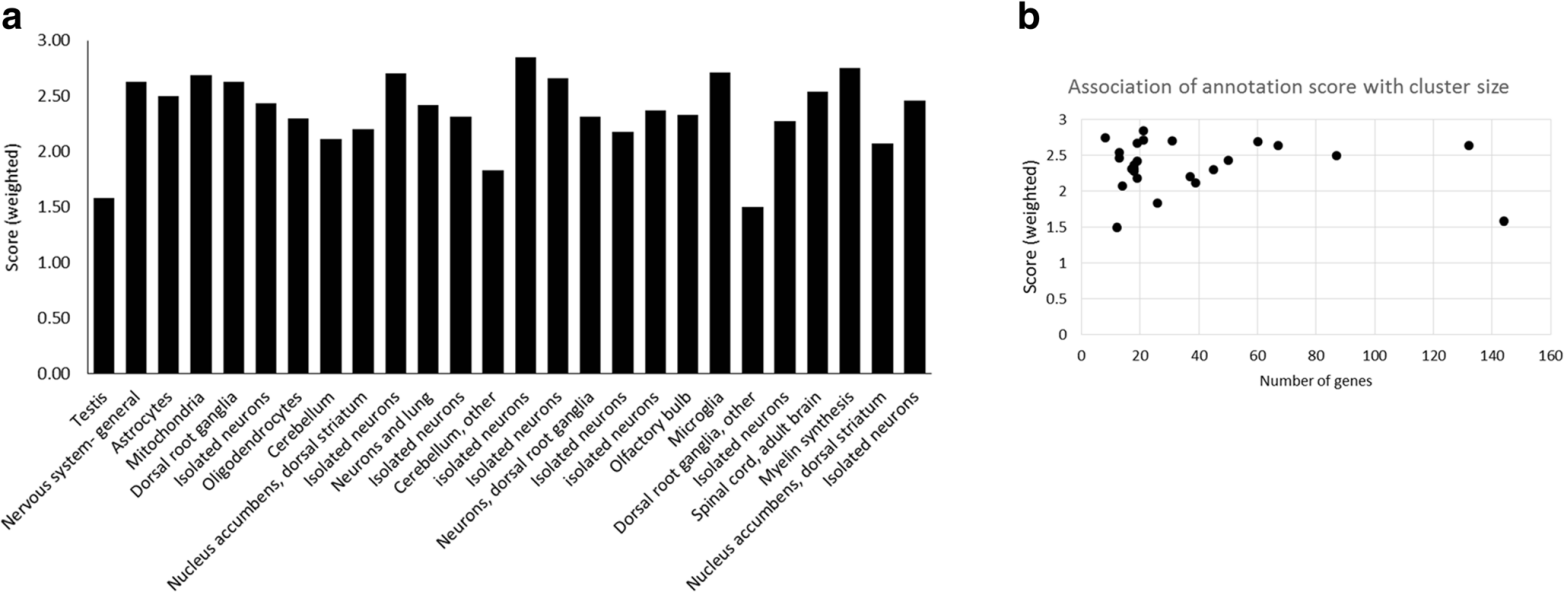
Gene ontology annotation scores for each gene cluster. **a** *Bar graph* showing overall weighted annotation scores for the gene clusters. Genes within each cluster were scored either 0 or 1 for each of the three gene ontology categories (GO cellular compartment, GO biological process, GO molecular function) depending on the absence or presence (no matter how minimal) of GO information. An average weighed score was calculated for each cluster (maximum score of 3 if each GO category had an entry for each gene in the cluster). Scores ranged from 1.50 (Cluster088, dorsal root ganglion) to 2.77 (Cluster045, isolated neurons). Clusters are shown in the order of cluster number (see Table 2). **b** Scatterplot of number of genes in a cluster compared with the weighed annotation score. The testis cluster was excluded from the graph because it had 10 times more genes than the next cluster (total 1628); the result when testis was included was similar

In addition to identifying well-annotated genes within the characterized expression clusters of the nervous system, we also identified genes that had little or no annotation in the most recent Affymetrix annotation file (October 2014), no mouse model and no associated human disease, and therefore were not represented in IPA pathway analysis. Some of the unannotated genes were identified as Affymetrix probe sets that had not been associated with a transcript; others were from the RIKEN curated set of complementary DNAs (cDNAs) [52] and no function or homology had been determined. There were also poorly annotated genes that encoded a recognized protein domain, but the specific function was not known. We utilized the gene ontology (GO) terms to examine the extent of gene annotation for all the clusters discussed above. Table 2 and Fig. 2a show a weighted annotation score for each cluster. Some clusters contained a high number of well-annotated genes, while others had many unannotated and minimally annotated genes. There was no association with cluster size since both the largest cluster (Cluster001) and some of the smaller clusters (for example Cluster088) were poorly annotated while other large clusters (for example, Cluster005) and small clusters (for example, Cluster091) were well annotated (Fig. 2b). All genes within the mitochondrial Cluster014 showed some degree of annotation, whereas one third of the genes in the testis Cluster001 had no GO term annotation. The clusters based on the nervous system showed variable degrees of annotation with the neuronal clusters well annotated and some of the region-specific clusters (cerebellum, dorsal root ganglia) poorly annotated.

**Table 2:** Degree of annotation for genes within each cluster

Cluster number	Description	*N*(0)	*N*(1)	*N* (2)	*N* (3)	*N* (genes)	Score (weighted)	% (0)	% (1)	% (2)	% (3)
1	Testis	541	236	212	639	1628	1.58	33	14	13	39
5	Nervous system—general	5	12	14	113	144	2.63	3	8	10	78
6	Nervous system—astrocytes	11	8	17	96	132	2.50	8	6	13	73
14	Mitochondria	0	6	15	66	87	2.69	0	7	17	76
27	Nervous system—dorsal root ganglia	3	4	5	48	60	2.63	5	7	8	80
30	Nervous system—neurons	7	5	7	48	67	2.43	10	7	10	72
35	Nervous system—oligodendrocytes	7	4	6	33	50	2.30	14	8	12	66
37	Nervous system—cerebellum	12	0	4	29	45	2.11	27	0	9	64
42	Nervous system—nucleus accumbens, dorsal striatum	8	2	3	26	39	2.21	21	5	8	67
45	Nervous system—neurons	3	0	2	32	37	2.70	8	0	5	86
48	Neurons and lung	1	6	3	21	31	2.42	3	19	10	68
53	Nervous system—neurons	1	5	0	13	19	2.32	5	26	0	68
56	Nervous system—cerebellum, other	6	1	1	10	18	1.83	33	6	6	56
59	Nervous system—neurons	0	1	2	23	26	2.85	0	4	8	88
60	Nervous system—neurons	1	1	2	17	21	2.67	5	5	10	81
66	Nervous system—neurons, dorsal root ganglia	0	3	7	9	19	2.32	0	16	37	47
73	Nervous system—neurons	3	2	1	11	17	2.18	18	12	6	65
77	Nervous system—neurons	1	2	5	11	19	2.37	5	11	26	58
82	Olfactory bulb	3	1	1	13	18	2.33	17	6	6	72
85	Nervous system—microglia	1	1	1	18	21	2.71	5	5	5	86
88	Nervous system—dorsal root ganglia, other	4	3	0	5	12	1.50	33	25	0	42
89	Nervous system—neurons	3	1	2	12	18	2.28	17	6	11	67
90	Nervous system—spinal cord, adult brain	2	0	0	11	13	2.54	15	0	0	85
91	Nervous system—myelin synthesis	0	1	0	7	8	2.75	0	13	0	88
96	Nervous system—nucleus accumbens, dorsal striatum	2	3	1	8	14	2.07	14	21	7	57
99	Nervous system—neurons	1	0	4	8	13	2.46	8	0	31	62

*N*(0), *N*(1), *N*(2), *N*(3) indicates the number of genes with 0, 1, 2 or 3 associated GO terms respectively. %(0), %(1), %(2), %(3) indicates the percentage of genes with 0, 1, 2 or 3 associated GO terms

### Cluster analysis allows functional annotation to be attributed to unannotated genes

Online Resource 6 lists the unannotated and poorly annotated genes of the nervous system clusters discussed above. The presence of a gene or transcript in a cluster indicates that its expression pattern is similar to those of other genes in the cluster and so its function is likely to be similar (‘guilt by association’ [9]). Therefore, the clustering process yielded further insight into function of the poorly annotated genes and this was validated by recent publications in some cases. Some examples are given below. For Cluster005, 17 genes had no or minimal (one GO category only) annotation. Expression of 2900011O08Rik (recently renamed *Minp*, migration inhibitory protein) has been detected specifically in the central and peripheral nervous system and enriched in the cerebral cortex. Knockdown of MINP protein in mouse resulted in accelerated radial migration and altered microtubule stability [53]. *Gdap1l1* showed minimal annotation and has previously only been linked to protein binding. Mutations in a similar human gene (*GDAP1*) cause Charcot-Marie-Tooth type 4A [54, 55], and this plus the result with mouse *Gdap1l1* suggests that human *GDAP1L1* is a candidate for neuromuscular disease. Despite having only one GO term, *Vsnl1* has been identified as a biomarker of Alzheimer disease in cerebrospinal fluid [56]. *A830039N20Rik* is unannotated with no associated GO terms. Analysis of gene expression in E15 stage mouse embryos identified expression in the developing brain/nervous system and visual system (retina inner and outer nuclear layers) by RNA in situ hybridisation (http://www.informatics.jax.org/marker/MGI:2445176). There are also several examples of genes with very minimal annotation, for example; *Tmem151a* and *Tmem179* (C14orf90) which have the attributed GO cellular component term “membrane”. Both show nervous system expression by RNA in situ hybridization according to the Jax database (http://www.informatics.jax.org/marker/MGI:2147713 and http://www.informatics.jax.org/marker/MGI:2144891). Clearly, the limited information available for these genes supports their role in the nervous system which was implied by their presence in Cluster005. There are several genes with no annotation at all in Cluster005; for example, *B230217C12Rik*. Their presence in this cluster highlights their potential role in nervous system form and/or function.

This also holds true for the other clusters. For example, Cluster006 (astrocyte) contains the minimally annotated gene *Olfml1* (olfactomedin-like 1)*.* Olfactomedin proteins are involved in the organisation and development of the nervous system [57]. A paralog of the *Olfm* gene, *Gldn* (gliomedin), functions in the formation and maintenance of nodes of Ranvier [58, 59] and the protein encoded by this poorly annotated gene may have a similar role. A knockout mouse model has now been made of the Cluster056 (cerebellum) gene *Frrs1l* showing partial preweaning lethality, limb grasping, abnormal gait, hyperactivity, abnormal behaviour, trunk curl, absent vibrissae and decreased grip strength, all neuronal phenotypes (www.mousephenotype.org/data/genes/MGI:2442704). A recent study showed SH2D5 protein (*Sh2d5* gene in Cluster096, nucleus accumbens and dorsal striatum) to be highly enriched in adult mouse brain and to associate with breakpoint cluster region protein (BCR), a regulator of Rho GTPases which is also highly expressed in the brain [60]. In situ hybridisation of the Cluster077 (neuronal) gene *A930009E05Rik* (also known as *Tmem178b*) showed exclusive expression in the nervous system (http://www.informatics.jax.org/marker/MGI:3647581). Additionally, the Cluster073 (neuronal) gene *Ctxn1* is expressed specifically in the brain and enriched in the cortex. In situ hybridisation reported expression in neurons of the cerebral cortex [61].

### Expression of unannotated genes in other datasets

We used four sources to validate the expression patterns of the poorly annotated genes: the Allen Brain Atlas (in situ hybridisation of RNA probes to C57BL/6J mouse tissues), MGI expression data (in situ hybridisation in mouse brain), human gene expression data from BioGPS and pig gene expression data from BioGPS. Many of the poorly annotated genes were not found in the human and pig, as they are identified only by mouse Ensembl IDs. Those that could be found showed concordance in all four datasets. For example, *Fbxo41* (mouse Cluster005 nervous system) was expressed in mouse brain regions, human brain and pig prefrontal cortex. *Baalc* (Cluster 006 Astrocytes) was expression in mouse brain regions, human brain and pig nervous system and thyroid. The results of this validation are summarized in Online Resource 7.

This highlight the benefits of this clustering analysis and through guilt by association allows us to predict that the unannotated genes for which little or nothing is known will be novel candidates for a role in maintenance of neuronal health with mutations/perturbations in expression linked to neurological disease.

### Neuronal clusters are perturbed in neurodegenerative disease

We were interested to see the impact of a disease state on the clusters we found. We therefore reanalysed the original samples with the addition of a dataset of adult mouse hippocampus 17 weeks after exposure to the ME7 prion infectious agent (Online Resource 2). The main tissue clusters (for example, testis, connective tissue, digestive tract) and functional clusters (protein synthesis, proliferation) were retained with very minor differences. However, genes in the original nervous system Cluster005 were distributed to a number of other clusters also showing high expression in the nervous system. Fifty-six percent were in ME7 Cluster014 and 24% were in ME7 Cluster055 and 057. In addition, some of the immune cell clusters were similarly disrupted with genes distributed to smaller clusters.

## Discussion

The rapid development of technologies facilitating large-scale genome and transcriptome analysis has led to the generation of a vast resource of data, with the capacity to offer new insights into the genetic organization and regulation of biological systems in health and disease. This has been particularly notable in the field of neurogenetics research (for examples, see [15–18, 62–68]). In this study, we used BioLayout *Express* ^3D^ [21] to interrogate publicly available databases of genome-wide expression results in the mouse. Unlike many other network analysis software tools, BioLayout *Express* ^3D^ employs an unstructured approach to cluster genes based on gene expression patterns across the sample set [9, 21]. It does not incorporate pre-existing knowledge of biological pathways and thus is able to identify previously undocumented relationships among genes. BioLayout *Express* ^3D^ enables the user to visualize complex relationships in two and three dimensions and cluster genes based solely on gene expression pattern. We were then able to identify clusters containing highly annotated genes and infer new functions/phenotypes of poorly annotated genes by guilt by association. Our overall goal was to identify novel genes as candidates for a role in nervous system health and function.

The level of a specific mRNA in a cell indicates the potential for the cell to make the encoded protein. To validate the relationship between RNA level and phenotypic outcome mediated by the protein product, we initially used two clusters of genes: the largest cluster identified in our analysis, Cluster001, associated with expression in testis, and a mitochondrial cluster (Cluster014). We showed that both of these clusters had corresponding phenotypes consistent with the expression pattern. The testis cluster was associated with reproductive phenotypes, including male sterility and the mitochondrial cluster was associated with known mitochondrial diseases. We also showed a high level of replication in a different mouse data set and in a smaller set of tissues from the pig, suggesting that the genes in the clusters are generally highly correlated. Addition of a neurodegenerative disease sample (ME7 prion infection) perturbed the nervous system and some immunological clusters, indicating that the disease state impacts on these groups of genes and providing potential insights into the disease process.

We then focused our analysis on distinct clusters of genes expressed in different cell types/regions of the nervous system. This analysis highlighted the differences between brain regions and cell types. High expression of some genes was common to all nervous system regions (general nervous system clusters in Table 1 and Online Resource 3). These included genes encoding proteins of synaptic and axonal compartments such as signalling molecules and receptor-mediated developmental guidance and patterning. Several regions were found to have specific gene expression signatures. For example, cerebellum was characterized by expression of genes involved in immunological and inflammatory responses [32] while the nucleus accumbens and dorsal striatum shared high expression of genes involved in protein kinase A signalling and mitochondrial permeability. These gene expression signatures were largely consistent with phenotypes generated in knockout mice and in humans with gene mutations. Approximately half of the genes identified in our BioLayout *Express* ^3D^ analysis clusters had a characterized knockout mouse model. The top-associated knockout mouse phenotype for the majority of clusters was linked to the nervous system, in particular, the synapse, reflecting the established role of the synapse in nervous system form and function (reviewed in [35]). We noted that not all clusters showed specifically neuronal phenotypes when the genes were knocked out in the mouse. Three clusters were enriched for behavioural phenotypes (Cluster053, Cluster027 and Cluster042), two clusters showed vascular and respiratory phenotypes (Cluster048 and Cluster045) and two small clusters contained genes related to gastrin release and obesity (Cluster089 and Cluster096). All of these can be linked back to the nervous system. For example, the brain controls appetite and food intake [69]. Additionally, a small number of mouse knockouts lacked any overt phenotype, which could be attributable to redundancy between different members of the same gene family, exemplified by the Cluster005 gene *Brsk1*. Knockout mice were viable and fertile with no overt phenotypic abnormalities (Online Resource 5). However, double *Brsk1*/*Brsk2* knockout mutants showed clear neurological phenotypes: minimal spontaneous movement, weak responses to stimulation and neonatal death [70]. Thus the role of these genes was only revealed when both were non-functional. Our approach to validation of our clusters has shown that generation of knockout models for other genes in the clusters will reveal novel genes that contribute significantly to neurological function.

Some markers considered definitive of specific cell types are not present in the corresponding clusters. There are a number of explanations for this. Firstly, many classic antibody-based markers of cell type have different names from the gene names. For example, the gene encoding the microglia marker IBA1 is *Aif1* in the current annotation. Secondly, some markers used to identify microglia in the brain are also found in macrophages, such as F4/80 (encoded by *Emr1*). Since we have several macrophage subsets in our analysis, these genes do not fall into the microglia cluster but into the main macrophage clusters. Thirdly, some of these markers have unique expression patterns, which are not correlated with the expression of any other gene at the threshold correlation coefficient value used. For example, the three *Csf1r* probe sets do not cluster with any other probe sets because of the unique expression pattern of *Csf1r* which includes expression in placenta. Finally, if these markers have low expression in the tissues analysed they would have been excluded by our filtering process.

We have previously shown that the level of gene annotation is associated with the intensity with which the tissue or function has been studied, often a reflection of whether the gene is tissue- or function-specific, when it is likely to be well annotated, or more ubiquitous and likely to be minimally annotated [9, 13]. Thus, the mitochondrial Cluster014 was well annotated, as was the myelin Cluster091 and the clusters highly expressed in neurons. These cell types and functions have been extensively examined for many years and it is not surprising that most genes in these clusters are well understood. In contrast, several clusters were poorly annotated, including testis Cluster001, dorsal root ganglia Cluster088 and cerebellum Cluster037 and 056. The testis has an extensive transcriptome of approximately 20,000 genes, with many novel splice variants, alternative promoter usage and long non-coding RNA species (see, for example, [71–73]). We found that one third of genes in the main testis cluster had no annotation, consistent with this extensive and novel transcriptome. The lack of annotation for the transcriptomes in the cerebellum and dorsal root ganglia suggests that these regions also have novel transcripts which may be a rich source of candidate genes for neurological conditions.

Importantly, this study enabled us to identify a large subset of genes with minimal or no GO annotation; some of which we have shown through the MGI Jax database, the Allen Brain Atlas, and human and pig expression data to be exclusively expressed within the nervous system and others linked to human disease. Presence of unannotated genes in a cluster indicates an expression pattern similar to that of the genes encoding proteins of known function in the same cluster and suggests that these genes are highly likely to represent novel candidates for roles in the regulation of form and/or function of the mammalian nervous system.

## Conclusions

Taken together, the phenotypes, pathways and GO term annotation associated with genes expressed in distinct cell types and region-specific clusters of the nervous system highlight the powerful contribution of such an extensive array of genes to the maintenance of neuronal homeostatic mechanisms. Importantly, we identified a large number of genes in each of the clusters which had minimal functional annotation. From the analyses reported in this study, we can now attribute putative functions to these poorly annotated genes, as we know the function of the genes they co-express with. These novel genes are likely to have important roles in regulating form and/or function of the mammalian nervous system. Further analysis of these genes, for example, through creating genetically modified mice and synthesizing and characterizing recombinant proteins, should reveal similar functions and phenotypes to other genes in the same cluster and consolidate their value as candidates for human neurological disorders.

## Electronic supplementary material

Online Resource 1 List of tissue samples used in the initial clustering analysis (DOCX 28 kb)

Online Resource 2 Supplementary experimental methods—validation datasets (DOCX 19 kb)

Online Resource 3 List of genes associated with each cluster (XLSX 48 kb)

Online Resource 4 List of human diseases associated with genes in each clusters (XLSX 45 kb)

Online Resource 5 List of knockout mouse and associated phenotypes associated with genes in each of the clusters (XLSX 340 kb)

Online Resource 6 List of unannotated/minimally annotated genes associated with each of the clusters (XLSX 20 kb)

Online Resource 7 Expression of poorly annotated genes in other datasets (XLSX 17 kb)
