# Supplementary material for: Analysis of gene expression in the nervous system identifies key genes and novel candidates for health and disease
Source: Neurogenetics. 2017 Feb 11;18(2):81–95. doi: 10.1007/s10048-017-0509-5 (PMC5359387; doi:10.1007/s10048-017-0509-5)
Supplement: Supplementary file 1 — List of tissue samples used in the initial clustering analysis (DOCX 28 kb) [file 10048_2017_509_MOESM1_ESM.docx]

**Analysis of gene expression in the nervous system identifies key genes and novel candidates for health and disease**

Neurogenetics

Sarah M Carpanini, Thomas M Wishart, Thomas H Gillingwater, Jean C Manson and Kim M Summers

Corresponding author: Professor Kim M Summers The Roslin Institute and Royal (Dick) School of Veterinary Studies, University of Edinburgh, Easter Bush, Midlothian, EH25 9RG, UK; [kim.summers@roslin.ed.ac.uk](mailto:kim.summers@roslin.ed.ac.uk)

**Online Resource 1**. List of samples and GEO datasets IDs. Samples are given in the order of presentation in **Figure 1b**. All samples were collected from C57BL/6 mice, except where stated.

| **GEO DataSets Accession** | **Sex and age** | **Cell type** | **Reference** |
| --- | --- | --- | --- |
| GSM200695  GSM200696  GSM200697 | Unknown, 9.5 days post coitus | E9.5 head | [[1](#_ENREF_1)] |
| GSM200706  GSM200707  GSM200708 | Unknown, 13.5 days post coitus | E13.5 head | [[1](#_ENREF_1)] |
| GSM258651  GSM258652 | Male, 8-10 weeks | Dorsal root ganglia | [[2](#_ENREF_2)] |
| GSM258635  GSM258636 | Male, 8-10 weeks | Cerebral cortex | [[2](#_ENREF_2)] |
| GSM258637  GSM258638 | Male, 8-10 weeks | Prefrontal cortex | [[2](#_ENREF_2)] |
| GSM258735  GSM258736 | Male, 8-10 weeks | Olfactory bulb | [[2](#_ENREF_2)] |
| GSM258733  GSM258734 | Male, 8-10 weeks | Nucleus accumbens | [[2](#_ENREF_2)] |
| GSM258653  GSM258654 | Male, 8-10 weeks | Dorsal striatum | [[2](#_ENREF_2)] |
| GSM258617  GSM258618 | Male, 8-10 weeks | Amygdala | [[2](#_ENREF_2)] |
| GSM258671  GSM258672 | Male, 8-10 weeks | Hippocampus | [[2](#_ENREF_2)] |
| GSM570750  GSM570751  GSM570752 | Female, > 6 months | Adult hippocampus | [[3](#_ENREF_3)] |
| GSM258673  GSM258674 | Male, 8-10 weeks | Hypothalamus | [[2](#_ENREF_2)] |
| GSM258633  GSM258634 | Male, 8-10 weeks | Cerebellum | [[2](#_ENREF_2)] |
| GSM258765  GSM258766 | Male, 8-10 weeks | Spinal cord | [[2](#_ENREF_2)] |
| GSM241896  GSM241904 | Unknown, postnatal day 16 (S100β-EGFP F1 C57BL/6 X DBA mice) | Neurons, P16 | [[4](#_ENREF_4)] |
| GSM241908 | Unknown, postnatal day 26 (S100β-EGFP F1 C57BL/6 X DBA mice) | Neurons, P26 | [[4](#_ENREF_4)] |
| GSM241912  GSM241914  GSM241926 | Unknown, postnatal day 17 (S100β-EGFP F1 C57BL/6 X DBA mice) | Astrocytes, P17 | [[4](#_ENREF_4)] |
| GSM241919 | Unknown, postnatal day 30 (S100β-EGFP F1 C57BL/6 X DBA mice) | Astrocytes, P30 | [[4](#_ENREF_4)] |
| GSM387046  GSM387047  GSM387048  GSM387049 | Unknown, postnatal day 7 plus 10 days in culture with PDGF-AA (mouse strain not specified) | Oligodendrocyte precursors | [[5](#_ENREF_5)] |
| GSM387042  GSM387043  GSM387044  GSM387045 | Unknown, postnatal day 7 plus 6 days in culture with PDGF-AA then 4 days after withdrawal of PDGF-AA in the presence of triiodothyronine (mouse strain not specified) | Oligodendrocytes | [[5](#_ENREF_5)] |
| GSM258721  GSM258722 | Male, 8-10 weeks | Microglia | [[2](#_ENREF_2)] |
| GSM258701  GSM258702 | Male, 8-10 weeks | Peritoneal macrophages | [[2](#_ENREF_2)] |
| GSM258693  GSM258694 | Male, 8-10 weeks | Bone marrow macrophages | [[2](#_ENREF_2)] |
| GSM258743  GSM258744 | Male, 8-10 weeks | Osteoclasts | [[2](#_ENREF_2)] |
| GSM258663  GSM258664 | Male, 8-10 weeks | Follicular B cells | [[2](#_ENREF_2)] |
| GSM258621  GSM258622 | Male, 8-10 weeks | Marginal zone B cells | [[2](#_ENREF_2)] |
| GSM258773  GSM258774 | Male, 8-10 weeks | CD4+ T-cells | [[2](#_ENREF_2)] |
| GSM258775  GSM258776 | Male, 8-10 weeks | CD8+ T-cells | [[2](#_ENREF_2)] |
| GSM258731  GSM258732 | Male, 8-10 weeks | Natural killer cells | [[2](#_ENREF_2)] |
| GSM258711  GSM258712 | Male, 8-10 weeks | Mast cells | [[2](#_ENREF_2)] |
| GSM258627  GSM258628 | Male, 8-10 weeks | Bone marrow | [[2](#_ENREF_2)] |
| GSM258767  GSM258768 | Male, 8-10 weeks | Spleen | [[2](#_ENREF_2)] |
| GSM258691  GSM258692 | Male, 8-10 weeks | Lymph nodes | [[2](#_ENREF_2)] |
| GSM258625  GSM258626 | Male, 8-10 weeks | Bone | [[2](#_ENREF_2)] |
| GSM258659  GSM258660 | Male, 8-10 weeks | Epidermis | [[2](#_ENREF_2)] |
| GSM258611  GSM258612 | Male, 8-10 weeks | Brown adipose | [[2](#_ENREF_2)] |
| GSM258613  GSM258614 | Male, 8-10 weeks | White adipose | [[2](#_ENREF_2)] |
| GSM258763  GSM258764 | Male, 8-10 weeks | Skeletal muscle | [[2](#_ENREF_2)] |
| GSM173005  GSM173006  GSM173007 | Male, 2-3 months | Heart (1) | [[6](#_ENREF_6)] |
| GSM258669  GSM258670 | Male, 8-10 weeks | Heart (2) | [[2](#_ENREF_2)] |
| GSM258689  GSM258690 | Male, 8-10 weeks | Lung | [[2](#_ENREF_2)] |
| GSM258761  GSM258762 | Male, 8-10 weeks | Salivary gland | [[2](#_ENREF_2)] |
| GSM258771  GSM258772 | Male, 8-10 weeks | Stomach | [[2](#_ENREF_2)] |
| GSM258677  GSM258678 | Male, 8-10 weeks | Small intestine | [[2](#_ENREF_2)] |
| GSM258675  GSM258676 | Male, 8-10 weeks | Large intestine | [[2](#_ENREF_2)] |
| GSM258747  GSM258748 | Male, 8-10 weeks | Pancreas | [[2](#_ENREF_2)] |
| GSM258615  GSM258616 | Male, 8-10 weeks | Adrenal gland | [[2](#_ENREF_2)] |
| GSM258749  GSM258750 | Male, 8-10 weeks | Pituitary | [[2](#_ENREF_2)] |
| GSM258779  GSM258780 | Male, 8-10 weeks | Testis | [[2](#_ENREF_2)] |
| GSM258753  GSM258754 | Male, 8-10 weeks | Prostate | [[2](#_ENREF_2)] |
| GSM258745  GSM258746 | Female, 8-10 weeks | Ovary | [[2](#_ENREF_2)] |
| GSM258789  GSM258790 | Female, 8-10 weeks | Uterus | [[2](#_ENREF_2)] |
| GSM258751  GSM258752 | Female, 8-10 weeks | Placenta | [[2](#_ENREF_2)] |
| GSM258787  GSM258788 | Female, 8-10 weeks | Umbilical cord | [[2](#_ENREF_2)] |
| GSM258709  GSM258710 | Female, 8-10 weeks | Non lactating mammary | [[2](#_ENREF_2)] |
| GSM258707  GSM258708 | Female, 8-10 weeks | Lactating mammary | [[2](#_ENREF_2)] |
| GSM258687  GSM258688 | Male, 8-10 weeks | Liver | [[2](#_ENREF_2)] |
| GSM258681  GSM258682 | Male, 8-10 weeks | Kidney | [[2](#_ENREF_2)] |
| GSM258623  GSM258624 | Male, 8-10 weeks | Bladder | [[2](#_ENREF_2)] |
| GSM258643  GSM258644 | Male, 8-10 weeks | Cornea | [[2](#_ENREF_2)] |
| GSM258679  GSM258680 | Male, 8-10 weeks | Iris | [[2](#_ENREF_2)] |
| GSM258685  GSM258686 | Male, 8-10 weeks | Lens | [[2](#_ENREF_2)] |
| GSM258759  GSM258760 | Male, 8-10 weeks | Retinal pigment epithelium | [[2](#_ENREF_2)] |
| GSM258757  GSM258758 | Male, 8-10 weeks | Retina | [[2](#_ENREF_2)] |

1. Hartl D, Irmler M, Romer I, Mader MT, Mao L, Zabel C, de Angelis MH, Beckers J, Klose J: **Transcriptome and proteome analysis of early embryonic mouse brain development**. *Proteomics* 2008, **8**(6):1257-1265.

2. Lattin JE, Schroder K, Su AI, Walker JR, Zhang J, Wiltshire T, Saijo K, Glass CK, Hume DA, Kellie S *et al*: **Expression analysis of G Protein-Coupled Receptors in mouse macrophages**. *Immunome Res* 2008, **4**(1):5.

3. Lunnon K, Teeling JL, Tutt AL, Cragg MS, Glennie MJ, Perry VH: **Systemic inflammation modulates Fc receptor expression on microglia during chronic neurodegeneration**. *J Immunol* 2011, **186**(12):7215-7224.

4. Cahoy JD, Emery B, Kaushal A, Foo LC, Zamanian JL, Christopherson KS, Xing Y, Lubischer JL, Krieg PA, Krupenko SA *et al*: **A transcriptome database for astrocytes, neurons, and oligodendrocytes: a new resource for understanding brain development and function**. *J Neurosci* 2008, **28**(1):264-278.

5. Emery B, Agalliu D, Cahoy JD, Watkins TA, Dugas JC, Mulinyawe SB, Ibrahim A, Ligon KL, Rowitch DH, Barres BA: **Myelin gene regulatory factor is a critical transcriptional regulator required for CNS myelination**. *Cell* 2009, **138**(1):172-185.

6. Dufour CR, Wilson BJ, Huss JM, Kelly DP, Alaynick WA, Downes M, Evans RM, Blanchette M, Giguere V: **Genome-wide orchestration of cardiac functions by the orphan nuclear receptors ERRalpha and gamma**. *Cell Metab* 2007, **5**(5):345-356.
