# Supplementary material for: Analysis of gene expression in the nervous system identifies key genes and novel candidates for health and disease
Source: Neurogenetics. 2017 Feb 11;18(2):81–95. doi: 10.1007/s10048-017-0509-5 (PMC5359387; doi:10.1007/s10048-017-0509-5)
Supplement: Supplementary file 2 — Supplementary experimental methods—validation datasets (DOCX 19 kb) [file 10048_2017_509_MOESM2_ESM.docx]

**Analysis of gene expression in the nervous system identifies key genes and novel candidates for health and disease**

Neurogenetics

Sarah M Carpanini, Thomas M Wishart, Thomas H Gillingwater, Jean C Manson and Kim M Summers

Corresponding author: Professor Kim M Summers The Roslin Institute and Royal (Dick) School of Veterinary Studies, University of Edinburgh, Easter Bush, Midlothian, EH25 9RG, UK; [kim.summers@roslin.ed.ac.uk](mailto:kim.summers@roslin.ed.ac.uk)

**Online Resource 2**

**Supplementary experimental methods: validation datasets**

1. FANTOM5 promoter based expression data from mouse.

Promoter level expression values were obtained using the Table Extraction Tool (<http://fantom.gsc.riken.jp/5/tet>). The analysis was performed using expression of the major promoter (p1@Genename) for each gene. Expression levels are provided as RLE normalised tags per million (tpm) {Forrest et al 2014}. Data were filtered to remove all genes with a maximum expression of less than 30 tpm. BioLayout *Express*^3D^ was run using a correlation coefficient threshold of 0.9 and an MCL inflation value of 2.2.There were 7,214 nodes and 1,730,162 edges in the analysis.

| **Tissue type** | **FANTOM5 Accession details** |
| --- | --- |
| Aortic SMC donor1 | Mouse Aortic Smooth Muscle cells, donor1 CNhs11297.11299-117B3 |
| Cerebellar astrocytes donor 1 | Mouse Astrocytes - cerebellar, donor1 CNhs13077.11708-123B7 |
| Cerebellar astrocytes donor 2 | Mouse Astrocytes - cerebellar, donor2 CNhs12076.11550-120C2 |
| Hippocampus astrocyte donor 1 | Mouse Astrocytes - hippocampus, donor1 CNhs12129.11709-123B8 |
| Hippocampus astrocyte donor 2 | Mouse Astrocytes - hippocampus, donor2 CNhs12077.11551-120C3 |
| Astrocytes donor 2 | Mouse Astrocytes, donor2 CNhs12078.11552-120C4 |
| Astrocytes donor 3 | Mouse Astrocytes, donor3 CNhs12107.11633-122C4 |
| CD19+ B cells donor 1 | Mouse CD19+ B Cells, donor1 CNhs13531.11856-125A2 |
| CD4+ T cells donor 1 | Mouse CD4+ T Cells, donor1 CNhs13509.11854-124I9 |
| CD8+ T cells donor 1 | Mouse CD8+ T Cells, donor1 CNhs13511.11855-125A1 |
| Cardiac myocytes donor 1 | Mouse Cardiac Myocytes, donor1 CNhs12355.11711-123C1 |
| Cardiac myocytes donor 2 | Mouse Cardiac Myocytes, donor2 CNhs12356.11729-123E1 |
| Cardiac myocytes donor 3 | Mouse Cardiac Myocytes, donor3 CNhs12353.11634-122C5 |
| Microglia donor 1 | Mouse Microglia, donor1 CNhs12629.11718-123C8 |
| Microglia donor 2 | Mouse Microglia, donor2 CNhs12634.11736-123E8 |
| Cortical neurons donor 1 | Mouse Neurons - cortical, donor1 CNhs12025.11724-123D5 |
| Cortical neurons donor 2 | Mouse Neurons - cortical, donor2 CNhs11947.11742-123F5 |
| Cortical neurons donor 3 | Mouse Neurons - cortical, donor3 CNhs12112.11647-122D9 |
| Spinal cord neurons dorsal donor2 | Mouse Neurons - dorsal spinal cord, donor2 CNhs12635.11738-123F1 |
| Spinal cord neurons dorsal donor3 | Mouse Neurons - dorsal spinal cord, donor3 CNhs12618.11643-122D5 |
| Hippocampal neurons donor 1 | Mouse Neurons - hippocampal, donor1 CNhs12133.11721-123D2 |
| Hippocampal neurons donor 2 | Mouse Neurons - hippocampal, donor2 CNhs12359.11739-123F2 |
| Hippocampal neurons donor 3 | Mouse Neurons - hippocampal, donor3 CNhs12110.11644-122D6 |
| Raphe neurons donor 1 | Mouse Neurons - raphe, donor1 CNhs12631.11722-123D3 |
| Raphe neurons donor 3 | Mouse Neurons - raphe, donor3 CNhs12619.11645-122D7 |
| Striatal neurons donor 1 | Mouse Neurons - striatal, donor1 CNhs12134.11723-123D4 |
| Striatal neurons donor 2 | Mouse Neurons - striatal, donor2 CNhs12360.11741-123F4 |
| Striatal neurons donor 3 | Mouse Neurons - striatal, donor3 CNhs12111.11646-122D8 |
| Substantia nigra neurons donor4 | Mouse Neurons - substantia nigra, donor4, tech_rep1 CNhs12612.11489-119E4 |
| Substantia nigra neurons donor5 | Mouse Neurons - substantia nigra, donor5 CNhs12614.11490-119E5 |
| Substantia nigra neurons donor6 | Mouse Neurons - substantia nigra, donor6 CNhs12643.11770-123I6 |
| Spinal cord neurons ventral donor 1 | Mouse Neurons - ventral spinal cord, donor1 CNhs12632.11725-123D6 |
| Spinal cord neurons ventral donor 2 | Mouse Neurons - ventral spinal cord, donor2 CNhs12638.11743-123F6 |
| Spinal cord neurons ventral donor 3 | Mouse Neurons - ventral spinal cord, donor3 CNhs12113.11648-122E1 |
| Schwann cells donor1 | Mouse Schwann, donor1 CNhs12507.11728-123D9 |
| Schwann cells donor2 | Mouse Schwann, donor2 CNhs12573.11746-123F9 |
| Hepatocytes donor 1 | Mouse hepatocyte, donor1 CNhs13078.11714-123C4 |
| Hepatocytes donor 3 | Mouse hepatocyte, donor3 CNhs12615.11637-122C8 |
| Hepatocytes donor 6 | Mouse hepatocyte, donor6 CNhs13090.11822-124F4 |
| Hepatocytes donor 8 | Mouse hepatocyte, donor8 CNhs13091.11824-124F6 |
| Lymph node | accessory axillary lymph node, adult CNhs10475.1063-29H9 |
| Bone | bone (os femoris), adult CNhs10483.56-12G2 |
| Cerebellum | cerebellum, adult CNhs10494.15-8B2 |
| Colon | colon, adult CNhs10468.36-18H7 |
| CMP rep 1 | common myeloid progenitor CMP, biol_rep1 CNhs12550.12122-128C7 |
| CMP rep 2 | common myeloid progenitor CMP, biol_rep2 CNhs12203.12125-128D1 |
| CMP rep 3 | common myeloid progenitor CMP, biol_rep3 CNhs12551.12128-128D4 |
| Corpora quadrigemina | corpora quadrigemina, adult CNhs10501.16-22A4 |
| Corpus striatum | corpus striatum, adult CNhs10487.19-21D8 |
| Cortex | cortex, adult CNhs10473.12-14D5 |
| Diencephalon | diencephalon, adult CNhs10482.20-12F2 |
| GMP | granulocyte macrophage progenitor GMP, biol_rep1 CNhs12206.12123-128C8 |
| GMP | granulocyte macrophage progenitor GMP, biol_rep2 CNhs12202.12126-128D2 |
| GMP | granulocyte macrophage progenitor GMP, biol_rep3 CNhs11928.12129-128D5 |
| Hippocampus | hippocampus, adult CNhs10478.13-16E8 |
| Intestine | intestine, adult CNhs10496.178-9A3 |
| Liver | liver, adult pregnant day01 CNhs10466.508-5B2 |
| Lung | lung, adult CNhs10474.28-22B1 |
| BMDM pool 1 | macrophage, bone marrow derived, pool1 CNhs11457.3560-170A1 |
| BMDM pool 2 | macrophage, bone marrow derived, pool2 CNhs11532.3632-171A1 |
| Mammary gland lactating | mammary gland, adult lactating day02 CNhs10480.595-22B6 |
| Mammary gland pregnant | mammary gland, adult pregnant day19 CNhs10476.588-5H2 |
| Medula oblongata | medulla oblongata, adult CNhs10477.17-12C2 |
| Olfactory brain | olfactory brain, adult CNhs10489.18-22I9 |
| ovary | ovary, adult CNhs10507.91-2I7 |
| Pancreas | pancreas, adult CNhs10486.34-16E4 |
| Pituitary gland | pituitary gland, adult CNhs10493.21-1G8 |
| Placenta | placenta, adult pregnant day10 CNhs10472.539-13I7 |
| Skin | skin, adult CNhs10492.30-1C3 |
| Spinal cord | spinal cord, adult CNhs10505.24-13C9 |
| Spleen | spleen, adult CNhs10465.25-2G2 |
| Stomach | stomach, adult CNhs10503.33-1H6 |
| Testis | testis, adult CNhs10504.57-7G5 |
| Thymus | thymus, adult CNhs10471.38-12B5 |
| Bladder | urinary bladder, adult CNhs10481.879-12E4 |
| Uterus | uterus, adult CNhs10509.92-27E5 |

1. Microarray based expression data from pig.

Microarray based expression data were from {Freeman et al 2012} and are available through BioGPS (<http://biogps.org>). Data are listed in the table; P number represents the pig from which the sample was taken; M and F indicate the sex of the pig. Datasets were normalised using the Affymetrix Expression Console. BioLayout *Express*^3D^ was run using a correlation coefficient threshold of 0.85 and an MCL inflation value of 2.2.There were 17,286 nodes and 1,730,162 edges in the analysis.

| Cortex_(prefrontal)-P3:F |
| --- |
| Cortex_(prefrontal)-P4:M |
| Hind_Brain_(medulla)-P3:F |
| Hind_Brain_(medulla)-P4:M |
| Cerebellum-P3:F |
| Cerebellum-P4:M |
| Pituitary-P1:F |
| Spinal_cord-upper-P2:F |
| Spinal_cord-upper-P3:F |
| Spinal_cord-lower-P2:F |
| Spinal_cord-lower-P3:F |
| Optic_nerve-P3:F |
| Optic_nerve-P4:M |
| Retina/sclera-P3:F |
| Retina/sclera-P4:M |
| Cornea/iris-P3:F |
| Trachea-P3:F |
| Trachea-P4:M |
| Lung_Parenchyma-P3:F |
| Lung_Parenchyma-P4:M |
| Tongue_(dermal_layer)-P3:F |
| Tongue_(dermal_layer)-P4:M |
| Salivary_glands_(submandibular)-P3:F |
| Salivary_glands_(submandibular)-P4:M |
| Oesophagus_(upper_third)-P3:F |
| Oesophagus_(upper_third)-P4:M |
| Oesophagus_(lower_third)-P3:F |
| Oesophagus_(lower_third)-P4:M |
| Stomach_(fundus)-P4:M |
| Stomach_(fundus)-P3:F |
| Stomach_(antrum)-P3:F |
| Stomach_(antrum)-P4:M |
| Pylorus_(smooth_muscle)-P3:F |
| Pylorus_(smooth_muscle)-P4:M |
| Duodenum-P3:F |
| Duodenum-P4:M |
| Jejeunum-P3:F |
| Jejeunum-P4:M |
| Ileum-P3:F |
| Ileum-P4:M |
| Caecum (apical )-P3:F |
| Caecum (apical )-P4:M |
| Caecum (mid)-P3:F |
| Caecum (mid)-P4:M |
| Colon_(proximal)-P3:F |
| Colon_(proximal)-P4:M |
| Colon_(distal)-P3:F |
| Colon_(distal)-P4:M |
| Rectum-P3:F |
| Rectum-P4:M |
| Pancreas-P3:F |
| Pancreas-P4:M |
| Bladder-P2:F |
| Bladder-P4:M |
| Kidney_(medulla)-P3:F |
| Kidney_(medulla)-P4:M |
| Kidney_(cortex)-P3:F |
| Kidney_(cortex)-P4:M |
| Liver-P3:F |
| Liver-P4:M |
| Gall_bladder-P3:F |
| Gall_bladder-P4:M |
| Ureter-P3:F |
| Ureter-P4:M |
| Skeletal_muscle_(leg)-P3:F |
| Skeletal_muscle_(leg)-P4:M |
| Skin_(head)-P4:M |
| Snout_tendon-P4:M |
| Heart-P4:M |
| Abdominal_aorta-P3:F |
| Abdominal_aorta-P4:M |
| Inferior_vena_cava-P4:M |
| Bone_marrow-P3:F |
| Bone_marrow-P4:M |
| Blood_1 |
| Blood_2 |
| Alveolar_macrophage_1 |
| Alveolar_macrophage_2 |
| BMD_macrophage_unstimulated |
| BMD_macrophage_7h_LPS |
| MD_macrophage_unstimulated |
| MD_macrophage_7h_LPS |
| Mesenteric_lymph_node-P3:F |
| Mesenteric_lymph_node-P4:M |
| Thymus-P3:F |
| Thymus-P4:M |
| Spleen-P3:F |
| Spleen-P4:M |
| Adrenal_gland_(cortex)-P3:F |
| Adrenal_gland_(cortex)-P4:M |
| Thyroid-P3:F |
| Thyroid-P4:M |
| Ovary-P3:F |
| Fallopian_tube-P3:F |
| Cervix-P3:F |
| Uterus-P3:F |
| Vagina-P3:F |
| Placenta:F |
| Epididymis-P4:M |
| Vas_deferens-P4:M |
| Vas_deferens-P4:M |
| Testis_(juvenile)-P4:M |
| Testis_(adult):M |
| Penis-P4:M |

1. Inclusion of a disease state in analysis

The analysis used the original dataset (**Online Resource 1**), with the addition of data for the transcriptome of the adult hippocampus of a mouse 17 weeks post infection with the prion agent ME7. Accession numbers for the datasets are GSM570750, GSM570751 and GSM570752 (available from GEO DataSets; https://www.ncbi.nlm.nih.gov/gds). The results of three experiments were averaged. Data were filtered to remove all genes with a maximum normalised intensity level of less than 100. BioLayout *Express*^3D^ was run using a correlation coefficient threshold of 0.9 and an MCL inflation value of 2.2.There were 17,014 nodes and 1,823,367 edges in the analysis.
